# *Aronia melanocarpa* Fruit Bioactive Fraction Attenuates LPS-Induced Inflammatory Response in Human Bronchial Epithelial Cells

**DOI:** 10.3390/antiox9090816

**Published:** 2020-09-02

**Authors:** Bong-Keun Jang, Jin-Woo Lee, Hyun Choi, Sung-Vin Yim

**Affiliations:** 1 Department of Medicine, Graduate School, Kyung Hee University, Seoul 02453, Korea; jbk@jbklab.co.kr; 2 Medical Science Research Institute, Kyung Hee University Medical Center, Seoul 02447, Korea; jwshsy@khmc.or.kr; 3 Department of Biomedical Science and Technology, Graduate School, Kyung Hee University, Seoul 02447, Korea; lionhyun@khu.ac.kr; 4 Department of Clinical Pharmacology and Therapeutics, College of Medicine, Kyung Hee University, Seoul 02447, Korea

**Keywords:** BEAS-2B cells, aronia bioactive fraction (ABF^®^), anti-inflammatory effects, cytokine, reactive oxygen species

## Abstract

To demonstrate the anti-inflammatory activity of *Aronia melanocarpa* fruit extract, human bronchial epithelial cells (BEAS-2B) were treated with lipopolysaccharide (LPS) and the effects of aronia bioactive fraction (ABF^®^), anthocyanin enriched extract from the fruit of *A. melanocarpa,* were evaluated. Following pretreatment with ABF^®^ at 10–25 µg /mL, BEAS-2B cells were exposed to LPS and the expression of inflammatory mediators (tumor necrosis factor [TNF]-α, interleukin [IL]-6, IL-8, regulated upon activation, normal T cell expressed and presumably secreted [RANTES], IL-1β, cyclooxygenase-2 [COX-2], and inducible nitric oxide synthase [iNOS]) was analyzed. In LPS-stimulated BEAS-2B cells, ABF^®^ pretreatment significantly decreased the mRNA expression of TNF-α, IL-6, IL-8, RANTES, IL-1β, and COX-2 at doses of 10 and 25 µg/mL. ABF^®^ also attenuated the secretion of TNF- α, IL-6, IL-8, and RANTES protein, as demonstrated by enzyme linked immunosorbent assay. Western blot analyses revealed the decreased expression of COX-2 and iNOS following ABF^®^ treatment. ROS production was decreased, and the cell cycle was arrested at the G0/G1 and S phases following ABF^®^ pretreatment. Our results suggest that ABF^®^ may have potential as a nutraceutical agent for the suppression of airway inflammation.

## 1. Introduction

Inflammation in the respiratory tract is associated with various respiratory diseases, including asthma, chronic obstructive pulmonary disease (COPD), cystic fibrosis, pneumonia, and upper respiratory tract infections (URIs) [1]. Respiratory diseases are the common causes of morbidity and mortality among children and the elderly [2]. Bronchial epithelial cells in the respiratory tract play a critical role in airway homeostasis. Inflammation in bronchial epithelial cells is the main pathogenic mechanism of pulmonary diseases. A hallmark of inflammation is the augmented secretion of pro-inflammatory immune mediators such as cytokines, chemokines, and prostaglandins (PGs) [3]. A systemic response follows the local secretion of pro-inflammatory cytokines and activation of immunocompetent cells in a physiologic microenvironment.

*Aronia melanocarpa* fruit (black chokeberries or Aronia berries) is a cherry-like edible berry of the plant family Rosaceae [4]. Because of their high polyphenol contents, particularly those of anthocyanins, aronia berries have garnered the attention of the public. Among various berries introduced in current markets in the world, the berry of *A. melanocarpa* was reported to have the highest content, 1.5% in fresh weight, of anthocyanin [5]. Presently, aronia is mainly cultivated in the northeastern European countries including Poland [5,6]. For many years, the antioxidant effects of plant polyphenols were believed to underlie their anti-inflammatory activities. However, more recently, polyphenols have been shown to regulate pro-inflammatory signaling pathways [7,8]. Thus, the aim of this study was to characterize the anti-inflammatory and antioxidant activities of anthocyanin enriched *A. melanocarpa* fruit extract ABF^®^, in BEAS-2B human bronchial epithelial cells as a potential nutraceutical agent for the treatment of pulmonary inflammatory diseases.

## 2. Materials and Methods

### 2.1. A. Melanocarpa Fruit Extracts and Chemicals

ABF^®^, partially purified extract powder by standardization of anthocyanins and the other polyphenols from the fruit of *A. melanocarpa,* was provided by JBKLAB (Gyeonggi-do, Korea). Anthocyanin content of ABF^®^ powder was standardized to around 16–18%. ABF^®^ was manufactured by extraction of the fruit of *A. melanocarpa* with 50% ethanol solution and by following the purification process for bioactive components. In the extract manufactured by the conventional extraction process, high amount of sugars like glucose and fructose were also present [9]. ABF^®^ is a bioactive fraction of *A. melanocarpa* fruit, separated from crude extract by removal process of those less bioactive sugars. ABF^®^ is anthocyanin enriched aronia extract and is expected to have better activity than conventional extracts.

Immortalized human bronchial epithelial cells (BEAS-2B) were maintained in bronchial epithelial cell medium (BEpiCM) (ScienCell, San Diego, CA, USA). Lipopolysaccharide (LPS) from *Escherichia coli* isotype 055:B5, dimethyl sulfoxide (DMSO), and 3-(4,5 dimethylthiazol-2-yl)-2,5-diphenyltetrazolium bromide (MTT) were purchased from Sigma-Aldrich (St. Louis, MO, USA). Enzyme-linked immunosorbent assay (ELISA) kits for tumor necrosis factor (TNF)-α, interleukin (IL)-6, and IL-8 were obtained from R&D Systems (Minneapolis, MN, USA), and the analyses were performed according to the manufacturer’s guidelines. A reverse transcription polymerase chain reaction (RT-PCR) kit was obtained from Promega (Madison, WI, USA). All other chemicals were of analytical grade (≥95%) and were purchased from Sigma-Aldrich. Inducible nitric oxide synthase (iNOS) antibody was acquired from Abcam (Cambridge, MA, USA). Primary antibodies against cyclooxygenase-2 (COX-2) (#4842) and β-actin (#4970), and horseradish peroxidase (HRP)-conjugated anti-rabbit immunoglobulin G (#7074) were obtained from Cell Signaling Technology (Beverly, MA, USA).

### 2.2. HPLC of ABF^®^

ABF^®^ was analyzed with HPLC in order to characterize anthocyanin, its main active component. HPLC sample solution of ABF^®^ was prepared with acidic methanol and detected at 520 nm for anthocyanin analysis. The other methods and analytical conditions for analysis were principally according to the method of Oszmianski et al. with some modification [10]. ABF^®^ was dissolved in 0.5% trifluoroacetic acid (TFA) in methanol and used for HPLC analyses after passing through a 0.22-μm syringe filter. The HPLC analysis was performed using an HPLC Agilent 1200 series (Agilent Technologies, Palo Alto, CA, USA) at 520 nm. A Zorbax SB C18 (250 × 4.6 mm, 5 μm) column (Agilent Technologies) was used for analyses at room temperature. Aqueous 0.5% TFA (A) and 0.5% TFA in 50% acetonitrile (B) were used as the mobile phase at a flow rate of 1.0 mL/min. A linear gradient was employed as follows: 0–15 min in 18–30% B; 15–18 min in 30% isocratic; 18–40 min in 30–50% B. The sample injection volume was 20 μL.

### 2.3. Cell Culture

BEAS-2B cells of human bronchial epithelial origin were provided by American Type Culture Collection (Rockville, MD, USA) and maintained in BEpiCM, with BEpiCGS (cell growth supplement, ScienCell) supplemented with antibiotic/antimycotic agents (ScienCell). Cells were grown and maintained at 37 °C and 95% humidity in an atmosphere of 5% CO_2_ and passaged with a split ratio of 1:3.

### 2.4. Cell Viability Assay

To investigate the possible cytotoxic effects of ABF^®^, cell viability was measured via the MTT assay. Briefly, cells were plated in triplicate at a density of 8000 cells/well in 96-well plates and incubated for 24 h. Then, the cells were treated with different concentrations of ABF^®^. The control group was treated with an equal volume of vehicle (0.01% phosphate-buffered saline [PBS]). After the cells were incubated for the desired duration, 20 µL MTT was added to achieve a final concentration of 2 mg/mL. After cells were incubated with MTT for 4 h at 37 °C, the supernatant in each well was carefully removed. Then, purple formazan crystals were dissolved in 200 µL DMSO, and the absorbance was measured at 570 nm using a microplate reader (Spark 10M; Tecan, Männedorf, Switzerland).

### 2.5. Quantitative Real-Time PCR (qRT-PCR)

Total RNA was extracted using a RNeasy Mini kit (Qiagen, Valencia, CA, USA), following the manufacturer’s protocol. The total RNA concentration was determined using a NanoDrop ND-1000 spectrophotometer (Wilmington, DE, USA) at an absorbance of 260/280 nm. First-strand cDNA was synthesized by RT-PCR in a 20-μL reaction mixture containing 1-μg extracted RNA, 5-μM random primers, 1-mM deoxynucleoside triphosphates, 1× reaction buffer, and 20 units of avian myeloblastosis virus reverse transcriptase (Promega, Madison, WI, USA). The individual sequences of the gene-specific primers are listed in Table 1. The qRT-PCR analyses were performed using a StepOnePlus real-time PCR System (Applied Biosystems, Foster City, CA, USA). The reaction mixture contained Power SYBR Green PCR Master Mix (Applied Biosystems) in a final volume of 20 μL, which included 1 µL of cDNA template, 2 µL of each primer (forward and reverse), 10 µL of Power SYBR Green PCR Master Mix, and 7 µL of nuclease-free water. PCR amplification was performed as follows: 95 °C for 10 min, then 40 cycles at 95 °C for 15 s for denaturation; and 60 °C for 1 min for primer annealing. When the cDNA expression of the target gene exceeded the cDNA expression of β-actin, the formula 2- (target gene-β-actin) was used to quantify the relative amounts of each cDNA.

### 2.6. Inflammatory Cytokine Determination in Cell Supernatant

BEAS-2B cells were pretreated with ABF^®^ for 6 h and then stimulated with LPS (1 μg/mL) for 48 h. The levels of inflammatory cytokines, including TNF-α, IL-6, IL-8, and RANTES, were measured in the culture supernatant using ELISA kits, according to the manufacturer’s instructions.

### 2.7. Western Blot Analysis

Cells were seeded at a density of 1 × 10^6^ /60-mm plate and, the following day, were pretreated with ABF^®^ (10 and 25 μg/mL) or 0.1% distilled water (*w*/*v*) as a vehicle control for 24 h. The cells were rinsed with cold PBS. The pellets obtained by centrifugation were resuspended in ice-cold extraction buffer [20 mM Tris-HCl (pH 7.5), 1 mM ethylene glycol tetraacetic acid, 2.5 mM sodium pyrophosphate, 150 mM NaCl, 1 mM β-glycerophosphate, 1 mM Na_2_ethylene diamine tetraacetic acid, 1 mM Na_3_VO_4_, 1 μg/mL leupeptin, 1% Triton, and 1 mM phenylmethylsulfonyl fluoride] and then placed in a tube on ice for 10 min. Subsequently, whole-cell lysates were centrifuged for 10 min at 12,000 rpm. The supernatants were then collected and used to determine total protein concentration using a BCA assay kit (Thermo Fisher Scientific, Waltham, MA, USA). Then, protein (20 μg) was loaded onto 8–12% sodium dodecyl sulphate polyacrylamide gels and subjected to electrophoresis, after which the proteins were electro-transferred to polyvinylidene fluoride membranes (GE Healthcare/Amersham Biosciences, Freiburg, Germany). Membranes were immersed in blocking buffer [5% non-fat dry milk in Tris-buffered saline (TBS) with 0.05% Tween-20 (TBS-T)] for 1 h at room temperature. Then, the membrane was incubated with antibodies against either COX-2 (dilution, 1:1000), iNOS (dilution, 1:500), or β-actin (dilution, 1:1000) overnight at 4 °C. Subsequently, the membrane was washed four times in TBS with Tween-20, and incubated with HRP-conjugated secondary antibodies (dilution, 1:2500) in TBS for 1 h. The chemiluminescent signal was detected by enhanced chemiluminescence (ECL; Thermo Scientific, Rockford, IL, USA) and an Amersham Imager 600 chemiluminescence imaging system (GE Healthcare, Piscataway, NJ, USA).

### 2.8. Intracellular Reactive Oxygen Species (ROS) Determination

Intracellular ROS generation was measured using the fluorescent dye 2’,7’-dichlorofluorescin diacetate (DCFH-DA) [11,12]. The DCFH-DA probe, a non-polar and non-fluorescent compound, is hydrolyzed by intracellular esterases to generate DCFH, which is retained in the cells. Oxidation of DCFH yields 2, 7-dichlorofluorescein (DCF), which can be detected by flow cytometry. In this assay, 5 × 10^5^ cells/well were seeded in 60-mm culture dishes, pretreated with ABF^®^ (10 and 25 μg/mL) for 2 h, and then treated with LPS (1 μg/mL). To test the effect of N-acetyl-cysteine (NAC) on H_2_O_2_ (100 μM)-induced ROS, the cells were pretreated with NAC (10 mM) for 2 h. Then, cells were washed once with PBS and treated with 20 μM of DCFH-DA at 37 °C in the dark for 30 min. Subsequently, intracellular ROS levels were examined immediately by flow cytometry.

### 2.9. Cell Cycle Analysis

Flow cytometry was used for cell cycle analyses. BEAS-2B cells were seeded (1 × 10^6^ cells/ 100-mm culture dishes) and pretreated with ABF^®^ (10 and 25 μg/mL) for 4 h. Then, cells were trypsinized to detach from the plate and rinsed twice with PBS before centrifugation. The cells were fixed with ice-cold 70% ethanol for 24 h and then stained with propidium iodide (PI) solution (50 μg/mL) and RNase A (10 µg/mL) (Bioneer, Daejeon, South Korea) at room temperature for 30 min. Approximately, 10,000 cell events were examined by flow cytometry (Becton Dickinson, Franklin Lakes, NJ, USA) using the BD CellQuest 6.0 software (version 6.0; BD BioScience, Germany).

### 2.10. Statistical Analyses

Data are presented as means ± standard deviations (SD). GraphPad Prism (version 5.01) was used for statistical analyses. Statistical significance was determined by one- or two-way analysis of variance (ANOVA) and a Bonferroni post-test was used for comparison of differences between control and ABF^®^ treatment. *p* ≤ 0.05 was considered statistically significant.

## 3. Results

### 3.1. HPLC of ABF^®^

**Figure 1:**
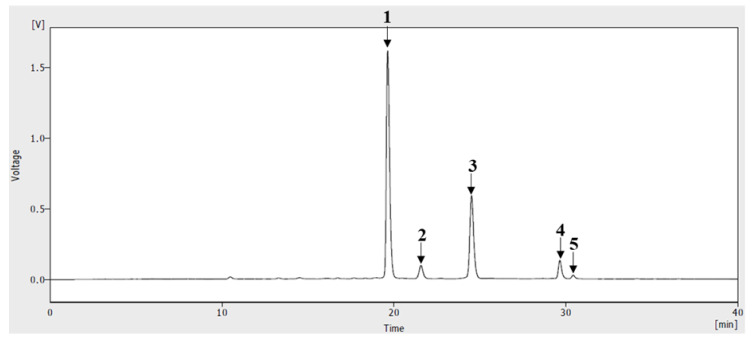
HPLC chromatogram of ABF^®^, anthocyanin enriched *Aronia melanocarpa* fruit extract at 520 nm. Peak numbers correspond to the following compounds: 1. cyanidin-3-galactoside; 2. cyanidin-3-glucoside; 3. cyanidin-3-arabinoside; 4. cyanidin-3-xyloside; and 5. cyanidin chloride.

Anthocyanin is assumed to be the richest and the most important bioactive constituents of ABF^®^, partially purified extract of *A. melanocarpa* fruit. In the HPLC analysis, performed to clarify the anthocyanin profile of ABF^®^, five peaks were observed at 520 nm (Figure 1). Each peak was chemically identified through a comparison with anthocyanin reference standard compounds. In anthocyanin analysis of ABF^®^, cyanidin and its four glycosides were observed. Four cyanidin glycoside were identified as cyanidin-3-galactoside, cyanidin-3-glucoside, cyanidin-3-arabinoside, cyanidin-3-xyloside and their ratio to total anthocyanins were 60.7%, 4.9%, 27.9% and 5.1% respectively. Ratio of aglycon cyanidin to total anthocyanins was only 1.4%. Total amount of cyanidin and cyanidin glycosides in ABF^®^ powder was 17.43 g/100 g (17.43%).

### 3.2. Cytotoxic Effects of ABF^®^ in BEAS-2B Cells

**Figure 2:**
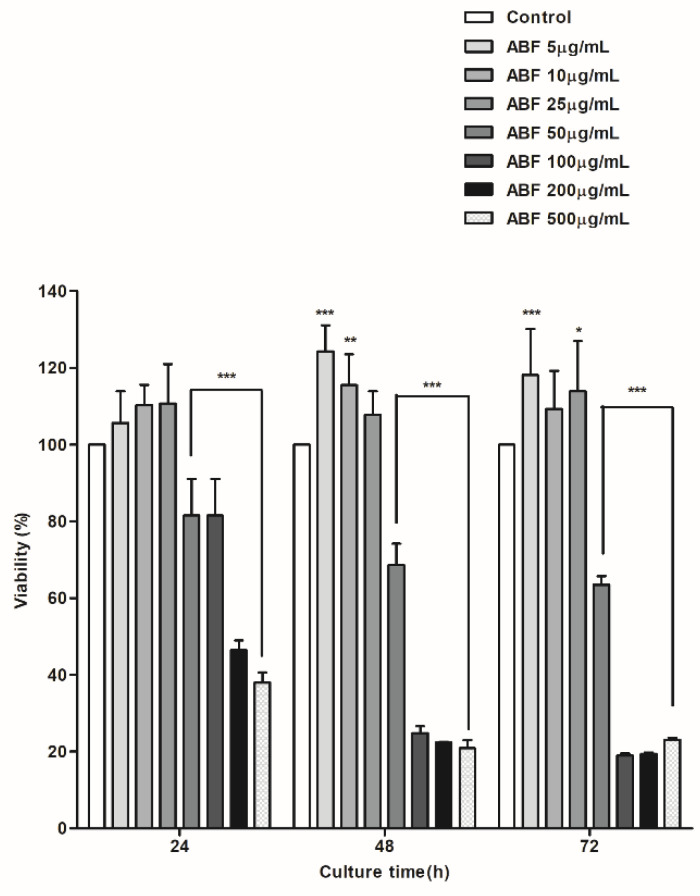
Cytotoxic effects of aronia bioactive fraction (ABF^®^) on BEAS-2B cells. Viability of BEAS-2B cells in response to different doses of ABF^®^. Cells were incubated with ABF^®^ for 24, 48, and 72 h and then used for a 3-(4,5 dimethylthiazol-2-yl)-2,5-diphenyltetrazolium bromide (MTT) assay. The values are presented as means ± standard deviation (SD) of three independent experiments. * *p* < 0.1, ** *p* < 0.05, *** *p* < 0.01 indicate significant differences as compared to the control cells.

To evaluate the selective cytotoxicity of ABF^®^, BEAS-2B cells were cultured in 96-well plates at a density of 8000 cells/well. Varying concentrations of ABF^®^ (5, 10, 25, 50, 100, 200, and 500 µg/mL) were added, and cells were cultured for 24, 48, and 72 h. After 24 h of treatment, no changes in cell viability were observed at concentrations up to 25 µg/mL; however, viability decreased by 19% at a concentration of 50 µg/mL (Figure 2). A concentration-dependent decrease in cell viability was observed following treatment with 50, 100, 200, and 500 µg/mL after 24, 48, and 72 h. ABF^®^ was not cytotoxic at 10 and 25 μg/mL (Figure 2), and these doses were therefore selected for use in subsequent experiments.

### 3.3. Effects of ABF^®^ on the mRNA Expression of Inflammatory Cytokines

**Figure 3:**
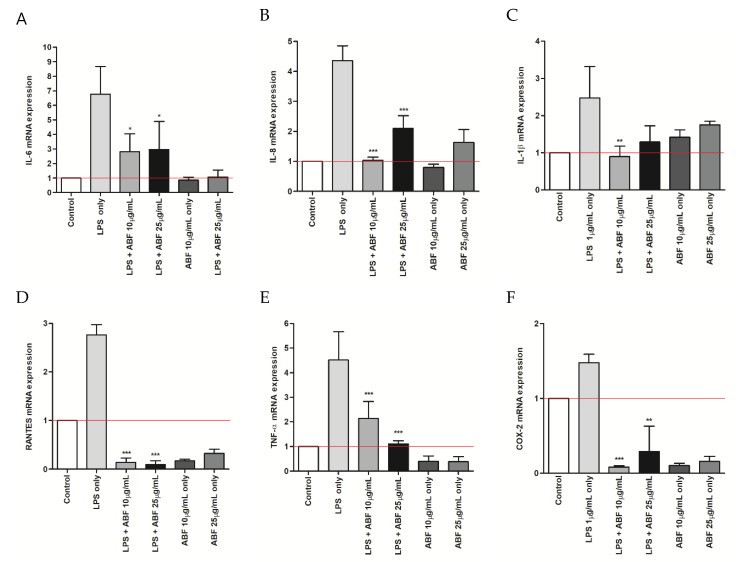
Effects of aronia bioactive fraction (ABF) on the lipopolysaccharide (LPS)-induced mRNA expression of interleukin (IL)-6 (**A**), IL-8 (**B**) and interleukin 1 beta (IL-1β) (**C**) regulated upon activation, normal T cell expressed and presumably secreted (RANTES) (**D**), tumor necrosis factor (TNF)-α (**E**) and cyclooxygenase-2 (COX-2) (**F**). BEAS-2B cells were pretreated with ABF (10 and 25 μg/mL) 6 h before treatment with LPS (1 μg/mL). After incubation for 6 h, the mRNA was evaluated by quantitative real time-polymerase chain reaction. The values are presented as means ± standard deviation (SD) of three independent experiments. * *p* < 0.1, ** *p* < 0.05, *** *p* < 0.01 indicate significant differences as compared to the LPS-treated cells.

We assessed the effect of ABF^®^ on the mRNA expression of inflammatory cytokines, including TNF-α, IL-6, IL-8, RANTES, and IL-1β in LPS-stimulated BEAS-2B cells. ABF^®^ suppressed the mRNA expression of TNF-α, IL-6, IL-8, RANTES, and IL-1β in LPS-stimulated BEAS-2B cells. COX-2 mRNA expression was also down-regulated following treatment with ABF^®^ (Figure 3).

### 3.4. Effects of ABF^®^ on the Protein Expression of Inflammatory Cytokines

**Figure 4:**
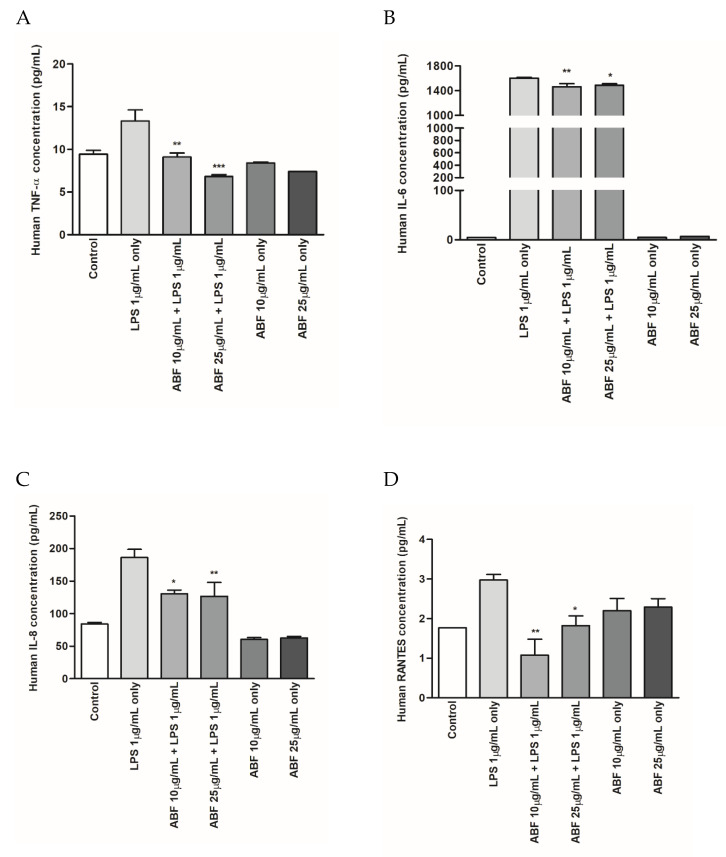
Effects of aronia bioactive fraction (ABF^®^) on inflammatory cytokines in lipopolysaccharide (LPS)-stimulated BEAS-2B Cells. Cells were pretreated with ABF^®^ (10 and 25 μg/mL) for 6 h and then treated with LPS (1 μg/mL) for 48 h. Cell culture supernatants were collected and tumor necrosis factor (TNF)-α (**A**), interleukin (IL)-6 (**B**), IL-8 (**C**), and regulated upon activation, normal T cell expressed and presumably secreted (RANTES) (**D**) production was assessed by enzyme-linked immunosorbent assay. Values are expressed as means ± standard deviation (SD) of triplicate experiments. * *p* < 0.1, ** *p* < 0.05, *** *p* < 0.01 indicate significant differences as compared to the LPS-stimulated cells.

The effects of ABF^®^ on the expression of inflammatory cytokines, including TNF-α, IL-6, IL-8, and RANTES, in LPS-stimulated BEAS-2B cells were evaluated using ELISA. Following the incubation of LPS-stimulated BEAS-2B cells with ABF^®^ (10 and 25 μg/mL), the culture supernatants were collected and used for subsequent cytokine analyses. The expression of inflammatory cytokines was markedly increased in the supernatant of LPS-treated cell cultures. However, ABF^®^ treatment significantly and dose-dependently inhibited TNF-α, IL-6, IL-8, and RANTES production in LPS-stimulated BEAS-2B cells (Figure 4).

### 3.5. Effects of ABF^®^ on LPS-Induced ROS Generation

**Figure 5:**
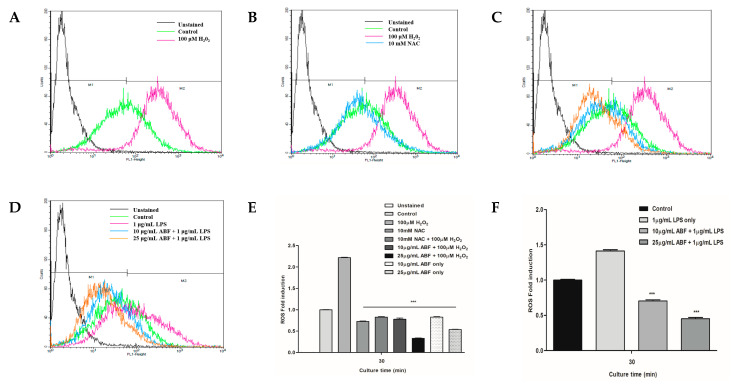
Effects of aronia bioactive fraction (ABF^®^) on lipopolysaccharide (LPS)-induced reactive oxygen species (ROS) generation in BEAS-2B Cells. Cells were pretreated with ABF^®^ (10 and 25 μg/mL) for 4 h before treatment with LPS (1 μg/mL) for 6 h. ABF^®^ attenuated LPS-induced ROS production in BEAS-2B cells. The values are presented as means ± standard deviation (SD) of three independent experiments. *** *p* < 0.01 indicate significant differences as compared to the LPS-treated cells. (**A**) control and 100 μM H2O2 treated cells; (**B**) Control, 100 μM H2O2 and 10 mM NAC treated cells; (**C**) Control, 10 μg/mL ABF and 25 μg/mL ABF treated cells; (**D**) Control, 10 μg/mL ABF + 1 μg/mL LPS and 25 μg/mL ABF + 1 μg/mL LPS treated cells; (**E**) ROS induction; (**F**) ROS induction and ABF’s effects under LPS stimulation.

To investigate the effects of ABF^®^ on ROS production, we assessed the ability of ABF^®^ to regulate ROS production following LPS stimulation. ROS production was significantly increased in LPS-stimulated cells, compared with that of the vehicle control. However, ABF^®^ treatment dose-dependently decreased ROS production in LPS-stimulated BEAS-2B cells, compared with that of the LPS-treated group (Figure 5).

### 3.6. Effect of ABF^®^ on Induction of Cell Cycle Arrest

**Figure 6:**
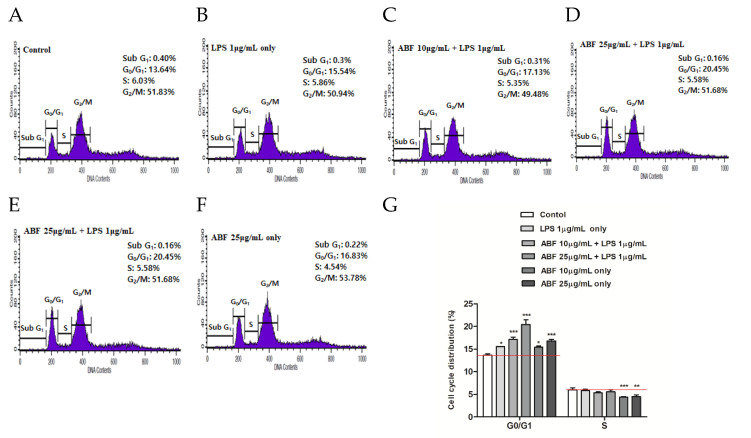
Induction of cell cycle arrest by aronia bioactive fraction (ABF^®^). Cells were pretreated with ABF^®^ (10 and 25 μg/mL) for 6 h and then treated with lipopolysaccharide (LPS) (1 μg/mL) for 24 h. Cell cycles of the control and ABF^®^-pretreated cells were analyzed by flow cytometry. (**G**) Changes in the G0/G1 and G2/M phases of ABF^®^-pretreated cells were compared with those of control cells. The values are presented as means ± standard deviation (SD) of three independent experiments. * *p* < 0.1, ** *p* < 0.05, *** *p* < 0.01 indicate significant differences as compared to the control cells. (**A**), Control; (**B**), LPS 1 μg/mL only; (**C**), ABF 10 μg/mL + LPS 1 μg/mL; (**D**), ABF 25 μg/mL + LPS 1 μg/mL; (**E**), ABF 10 μg/mL only; (**F**), ABF 10 μg/mL only.

Distribution of the cell cycle among LPS-stimulated BEAS-2B cells was assessed using flow cytometry. Figure 6 shows the relative ratios of cell cycle stages following ABF^®^ treatment. Following ABF^®^ treatment, the proportion of G0/G1 phase cells increased significantly from 13.64 ± 0.32% (control) to 17.13 ± 0.55% (10 μg/mL) and 20.45 ± 1.02% (25 μg/mL). Cell cycle arrest at G0/G1 phase was accompanied by a decrease in the proportion of the S-phase cell. The flow cytometry results showed that ABF^®^ inhibited BEAS-2B cell proliferation at the G0/G1 phase.

### 3.7. Effects of ABF^®^ on the Protein Expression of COX-2 and iNOS

**Figure 7:**
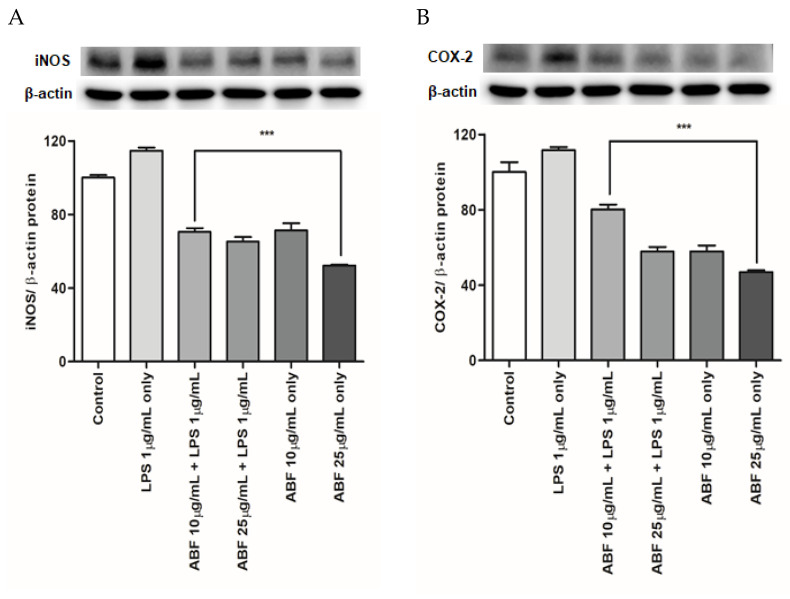
Effects of aronia bioactive fraction (ABF^®^) on inducible nitric oxide synthase (iNOS) (**A**) and cyclooxygenase-2 (COX-2) (**B**) protein expression in lipopolysaccharide (LPS)-stimulated BEAS-2B cells. ABF^®^ inhibited iNOS and COX-2 production (Figure 6). iNOS and COX-2 protein levels were confirmed by western blot. Expression of iNOS and COX-2 was markedly increased by LPS treatment and significantly reduced following ABF^®^ (10 and 25 μg/mL) pretreatment. Values are expressed as means ± standard deviation (SD) of triplicate experiments. *** *p* < 0.01 indicate significant differences as compared to the LPS-stimulated cells.

COX-2 and iNOS protein levels were evaluated by western blot analysis. Protein expression of COX-2 and iNOS was markedly upregulated following LPS treatment. However, ABF^®^ effectively and dose-dependently suppressed the LPS-induced increase in COX-2 and iNOS protein expression (Figure 7). Compared with the LPS group, the LPS-induced COX-2 expression was decreased by 31 and 54% with ABF^®^ at 10 and 25 μg/mL, respectively, and the LPS-induced iNOS expression was reduced by 44 and 49% with ABF^®^ at 10 and 25 μg/mL, respectively. β-actin was used as an internal control.

## 4. Discussion

The airway epithelium is the first-line defense against allergens, inhaled dust, and microorganisms and plays a major role in local immune responses of the host [13,14]. Airway inflammation induces the secretion of various anti- and pro-inflammatory cytokines, including TNF-α, IL-1, IL-6, IL-8, INF-γ, TGF-β, and IL-10. It is also a major source of RANTES, which attracts inflammatory cells into the airway [15]. Consequently, modulation of inflammatory cytokine production, and RANTES, in response to a variety of stimuli has been implicated in the pathology of various airway diseases [13,16,17,18,19]. To our knowledge, we, for the first time, show that ABF^®^ can reduce the expression of inflammatory substrates and the production of inflammatory cytokines in response to LPS stimulation in human bronchial epithelial cells. The inhibitory effect of ABF^®^ on inflammatory protein expression may account for its anti-inflammatory role in the respiratory tract. Therefore, ABF^®^ demonstrates potential as an anti-inflammatory and antioxidant agent through the suppression of ROS generation, RANTES expression, and immunomodulatory cytokine production in LPS-stimulated BEAS-2B cells (Figure 3D and Figure 5).

The potential anti-inflammatory effects of aronia fruit extract or juice have been widely reported [20,21,22,23]. Aronia fruit extract has been shown to dose-dependently inhibit NO production in LPS-stimulated Raw 264.7 cells [24,25]. Additionally, TNF-α and IL-6 were identified as key cytokines that are released following the activation of macrophages, such as LPS-stimulated Raw 264.7 cells. Aronia fruit extract was shown to decrease TNF-α and IL-6 expression, which are secreted before inflammation in a concentration-dependent manner [24]. A study by Ohgami et al. showed that aronia fruit crude extract reduced NO, prostaglandin E_2_ (PGE_2_), and TNF-α levels in the aqueous humor [9].

During an inflammatory response, TNF-α induces the expression of IL-6, which is a key inflammatory cytokine and is induced by various stimuli, including LPS. LPS activates a variety of transcription factors and induces the expression of several genes during inflammation [26]. IL-6 is one of the major initiators of the acute response and plays an important role in mediating the immune response to inhibit chronic inflammation. Studies have shown that the level of IL-6 is increased in various inflammatory diseases, including allergies, asthma, and autoimmune disease [27,28]. IL-8 production has been reported in normal human bronchial epithelial cells in response to various infectious agents. IL-8 exerts chemotactic effects for T cells and neutrophils. These cytokines may contribute to the airway inflammation that occurs during infections [29]. The results of the present study demonstrate that ABF^®^ efficiently attenuated the LPS-induced expression of pro-inflammatory cytokines, including TNF-α, IL-6, IL-8, and RANTES at both the mRNA and protein levels (Figure 3 and Figure 4).

ROS are partially reduced oxygen metabolites with strong oxidizing properties. Although low ROS concentrations have positive effects on cell growth, survival, and function, high concentrations can have detrimental effects on somatic cells. ROS are toxic to tissues because they oxidize a variety of cellular components and damage DNA. At normal levels, ROS is a main signaling molecule that controls cell growth, cell–cell adhesion, senescence, differentiation, apoptosis, and tumorigenesis [30,31]. Chronic or prolonged ROS exposure has been shown to drive pathogenesis in chronic pulmonary inflammatory diseases [32]. ROS include superoxide anion (O^2−^), hydrogen peroxide (H_2_O_2_), hydroxyl radical (OH^−^), and hypochlorous acid (HOCl). Because ROS are continuously generated through cellular metabolism, they may have important functions in cell signaling and disease. However, their functions and properties are not fully understood. In addition, the other main sources of ROS are cytochrome P450 enzymes and NADPH oxidases in a variety of cells, particularly in phagocytes and endothelial cells [31,33], which play central roles in inflammation [30]. Our results demonstrate the intracellular ROS scavenging effect of ABF^®^ as compared to the untreated cells (Figure 5).

Cell proliferation and differentiation are tightly regulated by the cell cycle. Cell cycle progression involves distinct stages (G0/G1, S, and G2/M), and cell-cycle arrest is a common suppressor of cell proliferation. ABF^®^ significantly increased the proportion of cells in the G0/G1 phase in a concentration-dependent manner and concomitantly decreased the proportion of cells in the S phase (Figure 6). Thus, ABF^®^ inhibited the proliferation of BEAS-2B cells by arresting the cell cycle at the G0/G1 phases.

Macrophages in airway epithelial cells play an important role in host defense against a variety of noxious stimuli, including infectious agents [34]. However, abnormal activation of epithelial cells and macrophages has also been suggested to play pathogenic roles in various inflammatory diseases. This is due to the production of excessive amounts of pro-inflammatory mediators and cytokines, which eventually contribute to aggravating the inflammation [35]. The most common cause of airway epithelial cell and macrophage activation is LPS exposure, which is an important component of the outer membrane of gram-negative bacteria [36,37]. LPS-treated airway epithelial cells have been used successfully to model airway inflammation. Exposure of cells to LPS has been shown to increase the expression of inflammatory mediators, including NO and PGE2, and pro-inflammatory mediators such as COX-2 and iNOS [38,39]. In the present study, ABF^®^ inhibited the production of COX-2 and iNOS in airway epithelial cells (Figure 7). These results suggest that ABF^®^ may have potential as an anti-inflammatory food supplement or drug candidate.

## 5. Conclusions

In summary, our in vitro studies show that ABF^®^ can regulate airway inflammation by attenuating the production of pro-inflammatory cytokines in LPS-stimulated BEAS-2B cells. Moreover, the results of the in vitro studies provide clear evidence for the anti-inflammatory activity of ABF^®^, which attenuates ROS secretion and induces cell cycle arrest. These data provide the first experimental support for the therapeutic application of ABF^®^ against various inflammatory airway disorders.

## Figures and Tables

**Table 1:** Primer sequences and lengths of amplified templates for real-time quantitative polymerase chain reaction (PCR) (qPCR).

Target Gene	Accession Number	Sequence	Product (bp)
IL-6	NM000600.5	5′-GTGTTGCCTGCTGCCTTC-3′ 5′-AGTGCCTCTTTGCTGCTTTC-3′	194
IL-8	NM000584	5′-GACATACTCCAAACCTTTCCAC-3′ 5′- CTTCTCCACAACCCTCTGC-3′	160
RANTES	M21121	5′-TTTGCCTACATTGCCCGC-3′ 5′-TTTCGGGTGACAAAGACGACT-3′	370
TNF-α	NM000594	5′-ATCTTCTCGAACCCCGAGTG-3′ 5′-GGGTTTGCTACAACATGGGC-3′	51
IL-1β	NM000576	5′-TGATGGCTTATTACAGTGGCAATG-3′ 5′-GTAGTGGTGGTCGGAGATTCG-3	140
TGF-β1	NM000660.5	5-TGAACCGGCCTTTCCTGCTTCTCATG -3′ 5′-GCGGAAGTCAATGTACAGCTGCCGC-3′	152
COX-2	U04636	5′-CAAATCCTTGCTGTTCCCACCCAT-3′ 5′-GTGCACTGTGTTTGGAGTGGGTTT-3′	173
β-actin	NM001101	5′-GCGAGAAGATGACCCAGATC-3′ 5′-GGATAGCACAGCCTGGATAG-3′	77
